# Web-based alcohol screening and brief intervention for Māori and non-Māori: the New Zealand e-SBINZ trials

**DOI:** 10.1186/1471-2458-10-781

**Published:** 2010-12-22

**Authors:** Kypros Kypri, Jim McCambridge, John A Cunningham, Tina Vater, Steve Bowe, Brandon De Graaf, John B Saunders, Johanna Dean

**Affiliations:** 1 Centre for Clinical Epidemiology and Biostatistics, School of Medicine and Public Health, University of Newcastle, Callaghan, NSW, Australia; 2 Injury Prevention Research Unit, University of Otago, Dunedin, New Zealand; 3 Centre for Research on Drugs & Health Behaviour, Department of Public Health & Policy, London School of Hygiene & Tropical Medicine, London, UK; 4 Centre for Addiction and Mental Health, Toronto, Ontario, Canada; 5 Centre for Behavioural Research in Cancer, Melbourne, Victoria, Australia; 6 Centre for Youth Substance Abuse Research, University of Queensland, Herston, Queensland, Australia and Disciplines of Psychiatry and Addiction Medicine, University of Sydney, Sydney, NSW, Australia; 7 Research Psychologist, Newcastle, NSW, Australia

## Abstract

**Background:**

Hazardous alcohol consumption is a leading modifiable cause of mortality and morbidity among young people. Screening and brief intervention (SBI) is a key strategy to reduce alcohol-related harm in the community, and web-based approaches (e-SBI) have advantages over practitioner-delivered approaches, being cheaper, more acceptable, administrable remotely and infinitely scalable. An efficacy trial in a university population showed a 10-minute intervention could reduce drinking by 11% for 6 months or more among 17-24 year-old undergraduate hazardous drinkers. The e-SBINZ study is designed to examine the effectiveness of e-SBI across a range of universities and among Māori and non-Māori students in New Zealand.

**Methods/Design:**

The e-SBINZ study comprises two parallel, double blind, multi-site, individually randomised controlled trials. This paper outlines the background and design of the trial, which is recruiting 17-24 year-old students from seven of New Zealand's eight universities. Māori and non-Māori students are being sampled separately and are invited by e-mail to complete a web questionnaire including the AUDIT-C. Those who score >4 will be randomly allocated to no further contact until follow-up (control) or to assessment and personalised feedback (intervention) via computer. Follow-up assessment will occur 5 months later in second semester. Recruitment, consent, randomisation, intervention and follow-up are all online. Primary outcomes are (i) total alcohol consumption, (ii) frequency of drinking, (iii) amount consumed per typical drinking occasion, (iv) the proportions exceeding medical guidelines for acute and chronic harm, and (v) scores on an academic problems scale.

**Discussion:**

The trial will provide information on the effectiveness of e-SBI in reducing hazardous alcohol consumption across diverse university student populations with separate effect estimates for Māori and non-Māori students.

**Trial registration:**

Australian New Zealand Clinical Trials Registry (ANZCTR) ACTRN12610000279022

## Background

Hazardous alcohol consumption is a leading cause of mortality and morbidity in high and middle income countries and an increasing problem in low income countries [1,2]. In an era in which state controls on the availability of alcohol have dramatically decreased [3], effective interventions to reduce demand for alcohol, that are deliverable to many, are needed.

Screening and brief intervention (SBI), involving the systematic identification of people with hazardous alcohol consumption and the provision of brief advice on how to reduce this, is now accepted as a key plank of public policy to reduce alcohol-related harm in the community. A meta-analysis of opportunistic SBI, which examined the outcomes of 34 randomised controlled trials, revealed significant reductions in consumption and alcohol-related problems [4].

Web-based screening and brief intervention (e-SBI) has certain advantages over practitioner-delivered SBI: it involves little or no clinician contact and it can be conducted anonymously. Additionally, e-SBI may be more acceptable to many drinkers than a face-to-face intervention [5]. While various computerized methods for delivering SBI have been developed [6,7], there had, until recently, been no rigorous efficacy trials of these interventions published in the scientific literature [8].

### Hazardous alcohol use among university students

University students have been found to have considerably riskier drinking patterns than their non-student peers [9-11]. For example, in New Zealand, a random sample of students (response rate 82%) from one large public university were found to have a prevalence of hazardous drinking (a score of 8 or higher on the Alcohol Use Disorders Identification Test; AUDIT[12]), which was double that of their peers in the general population [9]. The prevalence of harmful drinking, as indicated by a score of 15 or higher on the AUDIT, was three times higher than that among their non-student peers.

### Previous research on e-SBI

On the basis of extensive development research conducted at a university student health service in New Zealand [13,14], a first randomised controlled trial involving 104 participants was conducted in 2002. Students who screened positive for hazardous drinking (AUDIT ≥ 8) at an initial assessment, conducted electronically in the reception area, were randomly assigned to a leaflet-only control group or to receive an assessment and personalised feedback intervention, delivered entirely via the Internet [15]. This took an average of 15 minutes and was completed during the waiting time to see medical staff. Participants were followed up after six weeks and again after six months. Relative to controls, those who received the intervention drank 26% less alcohol after six weeks and had 24% fewer problems six months later [16].

These promising results were confirmed in the next trial of e-SBI, in which 1,010 students were screened over a 3 week period in the reception area and 576 were randomised to intervention or control groups. Participants were re-assessed 6 and 12 months later, with 85% retention. The effects were similar to those of the first trial [17]. At six months, relative to controls, patients receiving e-SBI reported significantly lower drinking frequency (-21%), lower total consumption (-23%), and fewer academic problems (-24%). Encouragingly, intervention effects endured. At 12 months, significant differences in total consumption (-23%; equivalent to 3.5 standard drinks per week) and academic problems (-20%) remained, and AUDIT scores were 2.2 points lower than those of controls [17].

This reduction in AUDIT score was estimated to be equivalent to an absolute risk reduction of 9% (95% CI 3% to 14%) in diagnoses of alcohol abuse and dependence [18]. Assuming the program could be implemented with 50% of the New Zealand student population, this equates to 1,424 cases of alcohol use disorders prevented per year, a significant public health benefit. A clear limitation of the primary care based delivery of e-SBI is that in many universities the student health service does not provide healthcare to the majority of the student population. Accordingly, to realise the population-level benefits of e-SBI, a pro-active case finding approach would be required.

To address this limitation, a third trial was conducted at an Australian university, where we sought to determine whether an e-SBI program called THRIVE (Tertiary Health Research Intervention Via E-mail) could be delivered on the basis of a universal screening program, i.e., by-passing the primary care setting. In addition to circumventing the problems of interfacing with a busy primary care service, the approach takes advantage of the economy of scale that can be achieved with the Internet, making it possible to offer assistance to thousands of students at low cost, including many who would not routinely come into contact with health services of any kind.

We invited 13,000 17-24 year-old students to complete a web-based AUDIT and 7,237 responded [19]. A third (n = 2,435) scored in the hazardous/harmful range (≥ 8) and were randomised to THRIVE or screening alone, and 2,050 (80%) completed at least one follow-up assessment. Intervention, delivered immediately following the assessment, consisted of 10 minutes of web-based assessment and personalised feedback. After one month, participants receiving intervention drank significantly less often (-11%), smaller quantities per occasion (-7%) and consumed a lower volume of alcohol overall (-17%), than did controls. At six months, intervention effects persisted for drinking frequency (-9%) and volume of alcohol consumption (-11%) [20].

Overall, the effects seen in the New Zealand trials were replicated but were somewhat smaller in the THRIVE trial. Nonetheless, given the reach of the intervention delivered on the basis of universal screening, THRIVE has greater potential to produce a population effect than primary-care based delivery of e-SBI.

### The need for large effectiveness trials

We are at the stage when there is a pressing need for large effectiveness trials. There being several recent trials conducted among university students, however, much of the research has been conducted in conditions which would generalise poorly to practice, and, with one exception, a study whose results are yet to be published [21], there have been no large, multi-site effectiveness trials. Trialling the intervention across multiple sites has the advantages of testing the robustness of its effects across different student drinking cultures, which national surveys have shown to vary in levels of consumption [22] and exposure to alcohol outlets [23] and promotion [24].

In addition to being firmly rooted in the experience of three previous clinical trials, the proposed study is based on extensive experience in surveying university student drinking via the Internet. In 2005 a web survey (response rate 65%) was conducted with large random samples of students from six New Zealand university campuses [22]. The survey was repeated in 2007 at eight campuses (response rate 68%). New Zealand is now uniquely placed in having up-to-date national student alcohol consumption data (from the 2005 and 2007 student surveys) on which to base the proposed intervention.

### Responsiveness to Māori health

New Zealand was founded on the basis of a treaty between the indigenous (Māori) peoples and those, largely from Great Britain [as it was then], who were part of the colonial expansion of European peoples. The Treaty of Waitangi, signed in 1840, and recognised as the founding document of New Zealand, grants to Māori the rights and privileges of British subjects.

The alcohol research undertaken among New Zealand university students has been conducted within a Māori health or Treaty framework. Accordingly, we adopted an Equal Explanatory Power model [25] for the national student surveys. We sought to invite equal numbers of Māori and non-Māori students from each campus in order to maximize the explanatory power of the study for Māori, who have traditionally been poorly served by population surveys, despite bearing a considerably greater burden of alcohol-related harm than non-Māori [26]. We have maintained this approach in the trial design, described below.

## Aim

The aim is to determine the effectiveness of e-SBI for Māori and non-Māori university students in New Zealand.

## Methods/Design

### Design

**Figure 1:**
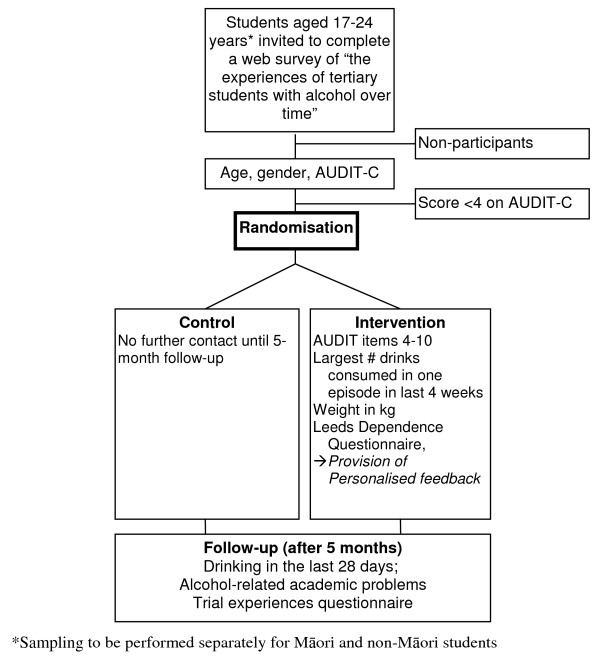
**e-SBINZ trial design**.

The study consists of two parallel, multi-site, double blind, individually randomised controlled trials. The trials are recruiting Māori and non-Māori students aged 17-24 years from seven of New Zealand's eight universities. Figure 1 shows the trial design.

### Sampling

In cooperation with each university administration, we have drawn random samples of undergraduates aged 17-24 years. This was done separately for Māori and non-Māori participants on the basis of self-reported ethnicity in university enrolment forms. The aim was to have equal numbers of Māori and non-Māori enrolled, however, that was impossible to achieve on some campuses, given low enrolments of Māori. In such campuses, we have invited all Māori in the requisite age group to participate and we increased the numbers of Māori invited from other universities accordingly.

### Recruitment

We have adapted a survey recruitment approach that has been used extensively and which has been described in detail elsewhere [27,28]. Four weeks after the start of the first semester random samples of students aged 17-24 years will be sent an e-mail containing a hyperlink to a web questionnaire. Up to three reminder e-mails will be sent in the following weeks to non-respondents.

### Instrument, randomisation, and intervention

Respondents will visit the study website and be presented with an introductory page followed by one page of five questions concerning (1) gender, (2) age, and the three items of the AUDIT-Consumption subscale [12]: (3) frequency of drinking; (4) number of standard drinks (defined as containing 10 g ethanol) consumed per typical drinking occasion; and (5) frequency of having six or more drinks per occasion. The AUDIT consumption subscale (AUDIT-C) is a valid screening tool, with specificity and sensitivity similar to the full AUDIT [29] and its psychometric performance has been previously evaluated in online studies with university students [30].

Upon clicking the submit button on this page, AUDIT-C scores will be calculated. Those who score ≥ 4 on the AUDIT-C will be randomly allocated by the web server to either the intervention or control group. The control group will be sent to a *Thanks *page at this point and advised that they will be contacted again by e-mail in second semester to complete a similar brief questionnaire.

The rationale for limiting the control group's alcohol questions to three items is that previous research shows that simply asking questions about alcohol consumption can act as intervention, producing reductions in self-reported drinking levels [31]. Indeed, such effects have been shown for the AUDIT alone [32].

Those in the intervention group will continue and be asked AUDIT items 4-10, and additional questions concerning the largest number of standard drinks consumed on one occasion in the last four weeks, the duration of the drinking episode in hours, and their body weight (for the purpose of estimating peak blood alcohol concentration). They will also complete the Leeds Dependence Questionnaire (LDQ; 10 items) [33]. The psychometric performance online of both the AUDIT and the LDQ has been confirmed in previous study with university students [34].

The intervention group will then receive personalised feedback consisting of: their AUDIT score and an LDQ score with an explanation of the associated health risk and information about how to reduce that risk; an estimated blood alcohol concentration (BAC) for the respondent's heaviest episode in the previous four weeks, with information on the behavioural and physiological sequelae of various BACs, and traffic crash relative risk; estimates of monetary expenditure per month; bar graphs comparing episodic and weekly consumption with that of other students and members of the general population of the same age and gender; and hyperlinks for help with drinking problems. Further web pages will be presented as options, offering facts about alcohol, tips for reducing the risk of alcohol-related harm, and where medical help and counselling support can be found. A demonstration version of the instrument can be viewed at http://ipru3.otago.ac.nz/limesurvey/.

### Follow-up

A high follow-up rate is clearly vital to the success of e-SBI trials and several approaches have been adopted to maximise this. In the second semester, approximately five months later, all participants will be invited to complete a brief on-line follow-up assessment, with the chance to win a $500 supermarket voucher or an Apple iPad. The assessment will include questions concerning the frequency of drinking and amount consumed per typical drinking occasion, all with a reference period of the last 4 weeks. In addition, participants will be presented with the five questions comprising the Academic Role Expectations and Alcohol Scale (AREAS) [35], plus questions concerning their subjective experience of the study.

### Blinding

Study participants will be unaware that they are involved in a trial, and that they have been randomised. They will have consented to complete a web survey of "the experiences of tertiary students with alcohol over time". The attention of the intervention group will not be drawn to the attempt to influence their behaviour. These decisions both serve to minimise bias relating to assessment reactivity and other aspects of study participation [36,37] and are also congruent with the aspiration to undertake the study in conditions as close as possible to those in which it would be routinely delivered.

### Sample size estimation

The same sample size will be sought separately for Māori and non-Māori students. The estimate is based on the following parameters and assumptions.

#### Sample size required for analysis at follow-up

We used as the basis for the estimate, the mean volume of alcohol reported at the 6 month follow-up THRIVE trial [38], namely 16.1 drinks (SD 15.9) for the control group and 13.6 drinks (SD 14.0) for the intervention group. Assuming a 5% level of significance, 80% power and a dispersion factor of 0.92 to reflect the skew in this measure [39], we would require 547 participants per group at follow-up in the proposed trial.

#### Attrition

Given the need for 1,094 cases (547 per group) to be analysed at follow-up, 1,563 individuals will have to be randomised at baseline (to either the intervention or control group), assuming attrition of 30% [1600/(1-0.3)]. This is considered realistic given the 6-month follow-up rate achieved in the THRIVE trial of 65% [38] and the fact that higher levels of participation were accomplished in our New Zealand research in this population (85% at 12 months) [17]. In addition, specific attention will be given to reducing attrition in the proposed study, including e-mail messages to non-respondents with two questions concerning the frequency of drinking and typical occasion quantity in the last four weeks--to be answered in the body of an e-mail message--from which modelling of attrition bias will be based [40].

#### Screening and consent

Given the need for 1,563 individuals to be allocated at baseline, we will seek consent for follow-up from 3,126 individuals, allowing for a 50% rate of screening negative or refusing consent for follow-up. This is informed by the proportion of New Zealand students who scored in the positive range in the national surveys (65%) [9].

#### Number to be invited to participate

The response rate in the THRIVE trial was 57%. Accordingly, on the conservative assumption that 40% agree to participate, we will need to invite 7,814 Māori and 7,814 non-Māori individuals. Given participation from seven universities we would therefore seek to invite 1,116 Māori and 1,116 non-Māori from each university.

### Outcomes

The primary outcomes are (1) volume of alcohol consumed per week, (2) frequency of drinking (occasions per week), (3) amount consumed per typical drinking occasion, (4) the proportion who exceed New Zealand recommended upper limits for: risk of acute harm (no more than 4 drinks per occasion for women; no more than 6 drinks per occasion for men); and (5) risk of chronic harm (no more than 14 drinks per week for women; no more than 21 drinks per week for men); and (6) scores on the AREAS [35].

### Analysis

The primary outcomes will be analyzed with negative binomial regression for panel data, using the Stata *xtnbreg *procedure [41]. For the proportions of students exceeding recommended upper limits we will use generalized linear mixed models with the *xtmelogit *procedure [42,43]. The results will be presented as readily interpretable risk ratios and odds ratios respectively. Participants will be analysed in the group to which they were randomized (intention to treat), with multiple imputation methods used for any missing follow-up data [44,45].

### Ethical approval

Ethical approval has been given by the Multi-region Ethics Committee, Ministry of Health (Ref: MEC/10/01/009).

## Discussion

A key attribute of a successful public health intervention is the extent it can be scaled up so that there are measurable impacts at a population level. This requires a series of stages in the supporting research. This trial has the potential to address a number of issues. First, if effects of the THRIVE trial [38], on which this trial is based, are replicated, there will be evidence that pro-actively delivered e-SBI is effective across a range of student cultures spanning two countries. This would be an important development given the largely efficacy evidence base on e-SBI to date. The findings would have clear applicability to university health promotion programs for this serious and pervasive health compromising behaviour.

Second, effect estimates will be produced separately for Māori and non-Māori. This would be the first study of a population intervention anywhere adopting the Equal Explanatory Power model on this scale for improving indigenous people's health. Critically, the findings could inform a decision as to whether to implement this intervention for Māori and non-Māori students in New Zealand.

Methodological strengths include the use of proven recruitment procedures, a validated screening instrument, up-to-date normative data, and a trial design that minimises the potential for assessment effects to bias estimates of intervention efficacy.

The major methodological challenges relate to recruitment and retention of participants. We plan to invite more than 15,000 individuals to participate and we are aware of no brief intervention trial ever having been conducted on this scale. Encouragingly, previous experience in the THRIVE trial in Australia [20] and surveys of New Zealand university students [22] show that using appropriate methods it is possible to recruit thousands of students within a few weeks and that the approach can be scaled up to include several university campuses.

The primary threat to validity will arise from any failure to retain a large proportion of participants for follow-up assessment. In the New Zealand primary care-based trials of e-SBI [16,17], in which recruitment was conducted face-to-face, retention was excellent: 85% at 12 months. In the THRIVE trial [38], however, where recruitment was online, only 65% of participants were retained at six months. Fortunately, there was no evidence of attrition bias, however, the potential for attrition to bias effect estimates increases as retention falls. Accordingly, we will adopt a method of obtaining a minimum response from those lost to follow-up with a view to estimating the degree of any attrition bias and potentially adjusting for it.

## Competing interests

The authors declare that they have no competing interests.

## Authors' contributions

KK conceived of and designed the study with input from JM, JC, JBS and JD. TV assisted in the co-ordination of the study and BDG conducted the web programming under the direction of KK. SB conducted the sample size estimation under the direction of KK. KK led the writing of the paper and all authors contributed to the writing or reviewed and approved the final draft.

## Pre-publication history

The pre-publication history for this paper can be accessed here:

http://www.biomedcentral.com/1471-2458/10/781/prepub
